# Supplementary material for: Perceptual learning of pitch provided by cochlear implant stimulation rate
Source: PLoS One. 2020 Dec 3;15(12):e0242842. doi: 10.1371/journal.pone.0242842 (PMC7714175; doi:10.1371/journal.pone.0242842)
Supplement: Attachment — Submitted filename: Response to Reviewers.docx [file pone.0242842.s001.docx]

Major Changes in Manuscript in response to comments:

Among the many changes the authors made in this revision, below we describe the more major changes made to improve the paper.

The authors reworked the methods and results sections of the revisions for flow and content, while adding significant paragraphs to the introduction and discussion to provide rationale, include relevant studies and improve the introduction and explanation of the metrics of spatial tuning and temporal integration.

The authors reworked the data analysis by re-quantifying masking proportional to the peak of the masking function in Figure 4 and normalizing the metrics by the average across conditions for each subject in Figure 6.

The authors recognize the significant statistical concerns and have worked to address those by going over data analysis and statistics, removing a figure, and designating planned vs exploratory statistical analyses.

Reviewer #1: The study examined individual variability in rate discrimination in cochlear implant users, and reported whether the variability can be attributed by spatial selectivity of neural excitation as well as the MPI functions. The paper also looked at the effect of training on rate discrimination. There were a lot of data, however, I found some of the results difficult to interpret because of several potential methodological issues. Introduction and methods can be improved by providing more in-depth rationale for the psychophysical measures chosen to be analyzed, choice of parameters, and the normalization procedures. Please find the more detailed comments below.

Re Introduction:

1) The rational of relating spatial tuning to rate discrimination needs to be elaborated. There is a brief discussion of this later in the Method section, but it should be moved here. Why do the authors think spatial tuning should be related to rate discrimination? Is it based on the assumption that sharper spatial tuning should indicate higher neural density? We know however, electrode position is also a strong predictor.

**Thank you for this comment, we have elaborated on the relationship of rate discrimination to spatial tuning in the introduction. The introductory paragraph that focuses on this concern begins on line 63 of the revision with “Hypothetically, stimulation rate discrimination…”.**

2) The authors used a general term, temporal tuning, to describe the effect of stimulation rate on thresholds. I think the term might be too broad. The mechanism undying the effect of rate on threshold might be completely different from that undying other temporal acuities, for example modulation sensitivity.

**Thank you for this comment, we have replaced references to temporal tuning with temporal integration or temporal precision in the revision.**

Methods:
3) Maybe I missed it but I don't see where the results of the masked thresholds using method of adjustment were reported. I wasn't quite sure that I understand why the masked thresholds were measured with method of adjustment at all.

**Thank you for this comment. We used a method of adjustment to quickly gauge masked thresholds across the entire array, but ultimately decided that the results did not contribute any insights beyond the forced-choice procedure. The description has been removed.**

4) I don't quite agree with the way that the masking patterns were quantified. First, I think that the amount of masking, rather than the absolute masking thresholds should be used. That is, the masked thresholds should be considered in terms of how much they have elevated from the unmasked values. You might have a very high masked threshold, but that does not necessarily mean that the masker has exerted a great amount of masking on this probe. Second, I don't quite follow how the masking functions were normalized. It appears that the masked threshold in % DR at the 0 position was subtracted from all data points. Based on methodology paper from McKay, the masking function (amount of masking against probe masker separation) should be divided by the peak of the function, rather than that at the 0 position. If the authors disagree with these methods, then I think the rationale should be provided.

**Thank you, we changed the quantification of masking to be amount of masking proportional to the peak of the masking function and this is reflected in Figure 4.**

5) It seems that each subject used a different electrode spacing for BP stimulation. I think it would be helpful to justify using different test parameters. I also wonder if this could have made it difficult to find an effect of stimulation mode?

**Good point, we addressed this as a limitation in the revision, with a paragraph added to the Discussion around line 483 beginning with “A limitation of the present study concerns how”.**

Results:

6) Figure2: it is unconventional to include results or stats in figure captions. Y label is not immediately clear and should be explained in methods.

**Thank you, we agree and have removed the stats from this figure caption (now Fig 5) and have explained the y label in the methods and results sections.**

7) The introduction section did not introduce the average detection thresholds as a part of the psychophysically derived metric. What was the rationale? I suspect that thresholds averaged across so many conditions, rates, location, and modes, would offer little information about the stimulation pattern or neural condition of the subjects. For example, we know thresholds at low versus high rates, and thresholds in MP versus BP stimulation would reveal completely different information about a stimulation site.

**Thank you for bringing this to our attention as we did not specifically introduce detection thresholds as the measure of spatial tuning in our introduction. We have elaborated on all our metrics including average detection thresholds in the introduction (the average meaning averaged across rates) beginning at line 68 with “Spatial tuning is quantified through forward-masked detection thresholds and average detection thresholds, which reflect multiple aspects of spatial tuning including electrode-neural geometry,…”.**

8) For some subjects and conditions, non-monotonic patterns were observed for the integration functions. These are unexpected, which makes me wonder, if the method of adjustment was accurate enough for estimating thresholds?

**Thank you for this comment. We have added a sentence in our methods at line 140 expanding on our method of adjustment saying “The subjects were instructed to loudness balance their detection…” and in the discussion at line 489 beginning with “Another possible limitation is some subjects exhibited a non-monotonic pattern in their detection thresholds which...”.**

9) Line 265-267: if the effect of subject was not significant, wouldn't it indicate less subject variability?

**Thank you for this comment. This does indicate less subject variability, which the authors phrased as a less consistent trend across conditions, which could easily be misunderstood. We have cleared this up around line 341 in the revision beginning with “In contrast to the other metrics, subject was not significant…”.**

10) Line 299-302: very interesting finding and I think it warrants some discussion

**Thank you for this comment, we have discussed this with a paragraph in the discussion starting at line 471 with “A significant interaction between stimulation mode and electrode location occurred for three of the measures: rate discrimination, forward-masked thresholds, and detection thresholds.”.**

11) Line 311-316: This paragraph is not clear. Considering rewording. Did the authors mean "as shown in Fig 3"?  What is a significant result at the individual level?

**Thank you for this comment, we have elaborated on the significance of the results and reworded this paragraph beginning around line 260 with “The significant interaction between subject, mode, and electrode location revealed…”.**

12) Figure 4: figure caption doesn't seem right.

**Thank you for this comment. This was supposed to be the caption for figure 5, which is now figure 2. The authors changed the figure caption to be more clear, and introduced the previous figure 5 (now figure 2 due to the reworking of the manuscript) earlier in the paper to be clearer in the differentiation of these 2 figures.**

13) The within-subject correlations may be done a little differently. For each measure, the data can be normalized to the average across conditions for each subject, thus removing between-subject variations. Correlations using differences between the apical and basal electrode certainly also removed the subject factor, but the within-subject effect of electrode condition was also removed. Also, maybe the authors would find the hypothesized relationships using thresholds at the higher base rates.

**Thank you for your comment on a better way to remove between-subject variations, we have implemented this. The significance of our results did not change, but our figure 6 now reflects this method of normalization. We also looked at relationships using rate discrimination thresholds at the higher base rates and found no difference in correlations.**

14) Line 352-360: These results were consistent with previous studies, and they indicate that the masked excitation patterns (related to spatial tuning) is negatively correlated with integration, so sharp spatial tuning and temporal tuning can't both indicate good neural condition. These need to be discussed in Introduction with more clearly laid out hypotheses.

**Thank you for this comment. It is a significant point. The introductory paragraph that focuses on this concern begins on line 63 of the revision with “Hypothetically, stimulation rate discrimination…”. The paragraph in the discussion that focuses on this concerned begins around line 447 of the revision with “In the present study, psychophysically derived metrics of spatial tuning and temporal…”.**

15) line 366-374: Do conclusions change if the post-training thresholds were considered for the various effects and correlations examined earlier?

**This is a very interesting point. The authors have address this at line 450 in the discussion beginning with “It is difficult to know how much of the correlations are driven by the plasticity of pitch and the underlying peripheral sensitivity.”.**

16) A general comment: some of the degree of freedom reported do not look right to me. Please check.

**Thank you, we rechecked the degrees of freedom and the analysis of variance for forward masked thresholds was done over another dimension (repetition) which we have corrected. We have adjusted the significances and degrees of freedom.**

17) Discussion: Line 430-432: please see my comments above regarding the relationship between spatial and temporal tuning - they are negatively correlated.

**You correctly pointed out that our results agree with the prevailing literature. We have discussed this in more detail around lines 367-372, 447-449, and 459-461 while still discussing why our results did not reach the same significance levels from lines 464-469.**

18) The current results showed that learning did not happen for higher base rates, but results from the earlier study from the same group showed that discrimination of extremely high rates can be learnt. This discrepancy should be discussed.

**Thank you for this comment, this is a very important point. We discuss this discrepancy in the Discussion paragraphs starting around line 428 with “The duration and amount of feedback seems to be a primary” and starting around line 435 with “Differences between the training protocol used in the present study and the training protocol used in Goldsworthy and Shannon, 2014, led to different amounts of training…”.**

Reviewer #2: This paper describes an experiment to better understand pitch perception in CI users based on rate of stimulation. Pitch perception was measured over several sessions, providing participants with practice using a cue that is not provided by their everyday CI processor. Other psychoacoustic measures – forward masking and multi-pulse integration -- were carried out to evaluate whether neural health was correlated with the potential for improved rate discrimination with practice. Data are consistent with the conclusion that there are practice effects for rate pitch discrimination, at least for low standard rates. Little or no learning occurred for 400 and 800 Hz (in contrast to the previous dataset), and no predictors of individual differences were found.

The topic of this research – rate discrimination abilities of CI users – is interesting and could have clinical implications. The experiment is sensible and appears to have been executed appropriately. The fact that no predictors were found does not diminish by interest in these data, as I think we have learned something by looking for such an association. The paper is well written at the level of the sentence and paragraph. My comments focus primarily on the overall organization, statistics, and interpretation of results.

Although the introduction lays out a clear argument for the research design, with hypotheses that are clearly focused on rate pitch discrimination, the results section does not maintain that organization. The results begin with the secondary measures, and rate discrimination only appears half-way through this section. There are also extensive statistical analyses of the secondary measure that do not appear to be directly motivated by the hypotheses. Overall, I was concerned about the number of statistical tests and the number of factors considered in this relatively limited dataset. Consider eliminating tests that are not clearly related to the hypotheses, or identifying those tests that were planned vs. exploratory. I strongly recommend consulting a statistician. Also consider including a rationale for testing difference stimulation modes and apical-vs-basal electrodes, with hypotheses and/or additional considerations.

Detailed comments

1) page 2, bottom: Based on this abstract, a reader would probably assume that rate pitch improved for all stimulation rates, including 400 and 800 Hz. This should be clarified.

**Thank you, we have clarified this by saying that there is an improvement at lower frequencies.**

2) line 32: Consider citing something that describes the controversy surrounding the maximum phase locking rate in humans. e.g., Verschooten, E., Shamma, S., Oxenham, A.J., Moore, B.C.J., Joris, P.X., Heinz, M.G., and Plack, C.J., The upper frequency limit for the use of phase locking to code temporal fine structure in humans: A compilation of viewpoints. Hear Res, 2019. 377: 109-121.

**Thank you, we have now cited this great paper and talked a little more about the debate in the field around line 34.**

3) line 44: I am not sure I would characterize this as “likely”. Consider changing this to “possible”.

**Thank you, we have done so.**

4) line 49: There is disagreement of number between “rates” and “it”.

**Thank you, this has been changed.**

5) Table 1: It is redundant to include age at implantation, age at test, and years of implant use. If you do retain that third column, “user” should be “use”.

**Thank you for this, we have changed it and have taken out years of implant use from the table since it was redundant.**

6) line 103: This wording is a little unusual. Consider changing, “an alternative forced-choice” to “a forced-choice”.

**Thank you, we changed this, but then took the wording out entirely when we consolidated this section through removing the method of adjustment forward-masked section.**

7) line 152: At this point I wondered how well these functions characterized the data.

**Thank you for this comment. We have expanded upon this in the methods around line 148 beginning with “Various equations were explored, and the fitted logistic equations provide the best compromise in terms of shape …”.**

8) line 167: Estimating thresholds based on an odd number of reversals introduces bias.

**Thank you for this point, we redid the data analysis with 4 reversals instead of three and came up with similar results, but, per your comment, chose to use an even number of reversals to avoid bias. The changes in F and p values in the rate discrimination section are due to this change, but there were no categorical changes in significance.**

9) line 199: Some of the text at the beginning of each section seems redundant with the methods. In any case, consider starting with the outcome of primary interest – rate pitch discrimination.

**Thank you, we changed the methods and results to have rate pitch discrimination be the first procedure introduced.**

10) line 316: Could there be order effects? I am not convinced that these data provide insight, as opposed to reflecting measurement noise.

**Thank you for this comment. Counterbalancing was done between test sessions which we have added at line 121 saying “Every measure was tested at two locations (apical, basal) for two stimulation configurations (bipolar, monopolar) with counterbalancing for all locations and configurations across test sessions.” That being said upon further study, we removed the figure showing individual subject, mode, and electrode interactions and explained why in the paragraph at line 260 beginning with “The significant interaction between subject, mode, and electrode location revealed that although there was not a group benefit for stimulation mode or electrode location, some individuals…”.**

11) line 347: At this point I became increasingly concerned about statistical power.

**Yes, thank you for pointing this out. According to your suggestion, we now have specified that this was an exploratory correlation of the proposed metrics.**

12) line 426: It was not clear to me how to interpret “removing across-subject variability”.

**We were not clear about how we removed across-subject variability. We are now normalizing to the average across conditions for each subject, thus removing between-subject variations. The authors changed the wording of this section and took this out instead explaining it in the results for planned correlation analyses beginning at line 362 and for exploratory correlations analyses at line 380.**

13) line 432: This study about integration and rate discrimination seems important, given that an association between these measures underlies the research design. Consider talking more about this dataset.

**Thank you for this comment, we have expanded our paragraph on metrics in the introduction to directly address the relationship between integration and rate discrimination at line 68 beginning with “Spatial tuning is quantified through forward-masked detection thresholds and average detection thresholds, which reflect…”. We have also expanded our results and discussion on the metric to talk more about the current literature and the relationship of the metrics to rate discrimination.**

14) line 468: Text in the introduction might lead a reader to assume that nothing like this has been done. Consider mentioning this study in the introduction.

**Thank you, this is a very important point for us to make in our introduction and we now do so at line 52 beginning with “Efforts to restore temporal fine structure to cochlear implant stimulation have demonstrated…”. We also have cited a review paper on plasticity and a few more studies in the discussion (lines 516-521).**

15) line 490: This seems to assume that learning would take place under the right conditions. I am not sure that is a valid assumption.

**Thank you, this is an important point. We have added some discussion about this to the last two paragraphs of the paper beginning at line 516. We agree that learning will not necessarily occur even under the right conditions, but that for the possibility of learning to occur, the right conditions and exposure/training must be presented. We base this on a few training studies including Goldsworthy and Shannon (2014).**

16) line 499: Consider specifying that this occurred for base rates of 100 and 200 Hz.

**Thank you, we have specified this at line 553.**
