# Supplementary material for: Perceptual learning of pitch provided by cochlear implant stimulation rate
Source: PLoS One. 2020 Dec 3;15(12):e0242842. doi: 10.1371/journal.pone.0242842 (PMC7714175; doi:10.1371/journal.pone.0242842)
Supplement: Attachment — Submitted filename: Response to Reviewers.docx [file pone.0242842.s002.docx]

November 6, 2020

Dear Dr. Ifat Yasin,

The authors thank you and the reviewers for the helpful comments provided for our paper throughout this revision process. In the current revision, we have addressed all comments – both specific and general – from both reviewers. Reviewer #1 had minor changes, which we addressed in the manuscript and comment on below. Reviewer #2 strongly recommended consulting a statistician. We consulted a statistician who provided two main recommendations.

The statistician’s first main recommendation was to clearly describe our data and intended statistical analysis in the methods section. The statistician commented that the strength of the collected data is that the data set contains repeated measures and was a full-factorial design, two things that were not clear in the original manuscript. The revision provides a clearer picture of the depth and fullness of the data set which supports the first level of hypothesis testing.

The statistician’s second main recommendation was to remove third order and higher considerations from the analyses. She commented that the depth of the data, having repeated measures and of full-factorial design, provides power to consider higher order interactions, but that doing so ultimately detracted from the analysis. As this was in line with the reviewer’s concerns, we fully agreed and consequently removed third-order and higher analyses from the manuscript.

Here we provide a structured account of manuscript changes:

I. Introduction: *In the previous revision*, the authors added rationale for considering stimulation modes and electrode locations and for considering metrics of spatial tuning and temporal integration. In this revision, the authors made minor word changes for clarity.
II. Methods: In this revision, we added an analysis subsection to the methods section (section II. C.) that delineates the experimental design and planned analyses.
III. Results: In this revision, the statistician recommended that we remove the 3^rd^ and higher order interactions from the statistical analysis for rate discrimination. The analysis of variance was conducted with second-order interactions; the categorical statements of significance have not changed from the prior submission.
IV. Discussion: *In the previous revision*, we added multiple paragraphs to the Discussion introducing the discussion, addressing limitations to the study, and discussing trends seen among metrics. In the current revision, we change some minor wording issues and added a few sentences at lines 488-494 addressing a trend among the measures.

**Below the author’s address specific reviewer comments:**

Reviewer #1: I recommend that the paper be accepted for publication - the paper has considerably improved after the revision and the authors were extremely responsive to my previous comments. The findings were of high important to the field. It is fascinating that rate discrimination is so trainable and the results provide strong evidence for using variable rates.

**Author response: Thank you very much!**

Just a few minor remaining issues:
Line 225: please provide rationale for using logarithmically transformed thresholds.

**Author response: Thank you, we have provided a reference (Micheyl et al. 2006) to the rationale for logarithmically transformed thresholds at lines 220-222 beginning with “All statistics were calculated…”.**

Legend of Figure 4: this seems to be the only place where normalization was described. A little more details are needed. Was proportion of percent dynamic range calculated in linear scale or log scale?

**Author response: We now describe the normalization in the results at line 305-307 beginning with “Thresholds were normalized in linear scale by dividing the forward masking…”. The normalization, or proportion of %DR was calculated in linear scale.**

Line 303: combine the two sentences

**Author response: Thank you, we have done so at line 316-318.**

Line 474: should be the "shallowest slopes"

**Author response: Thank you for this comment, we have changed this at lines 479 and 489.**

Line 482: needs a little more elaboration

**Author response: Thank you for this comment. We have added two sentences to the discussion section to elaborate on the possible explanation for the surprising trend that was seen. These sentences begin with “This trend is in agreement with Zhou and colleagues (2019) with the sites exhibiting the shallowest forward-masked thresholds slopes…” at lines 488-494.**

Reviewer #2: My primary comment on this manuscript is the same one I had on the previous version. I am concerned that the modest n does not support the extensive statistical analysis. It is problematic to run an ANOVA with a four-way interaction with n=7. I previously suggested that the authors consult a statistician, and I continue to believe that is the best way forward. A structured response to reviewer comments would also be helpful.

**Author response: Thank you for your comments. We have consulted with a statistician who indicated that her primary recommendation was to clearly describe our data and intended analysis in the methods section. The statistician commented that the strength of the collected data is that the data set contains repeated measures and was of full-factorial design, two things that were not clear in the original manuscript. The revision provides a clearer picture of the depth and fullness of the data set which supports the first level of hypothesis testing. The statistician seconded the concern with third and higher-order interactions, which we have removed.**
